# Supplementary figures and images for: Effects of Amnioreduction before Physical Examination-Indicated Cerclage on Pregnancy Outcomes: A Propensity Score Matched Study
Source: J Clin Med. 2023 Mar 24;12(7):2480. doi: 10.3390/jcm12072480 (PMC10095065; doi:10.3390/jcm12072480)

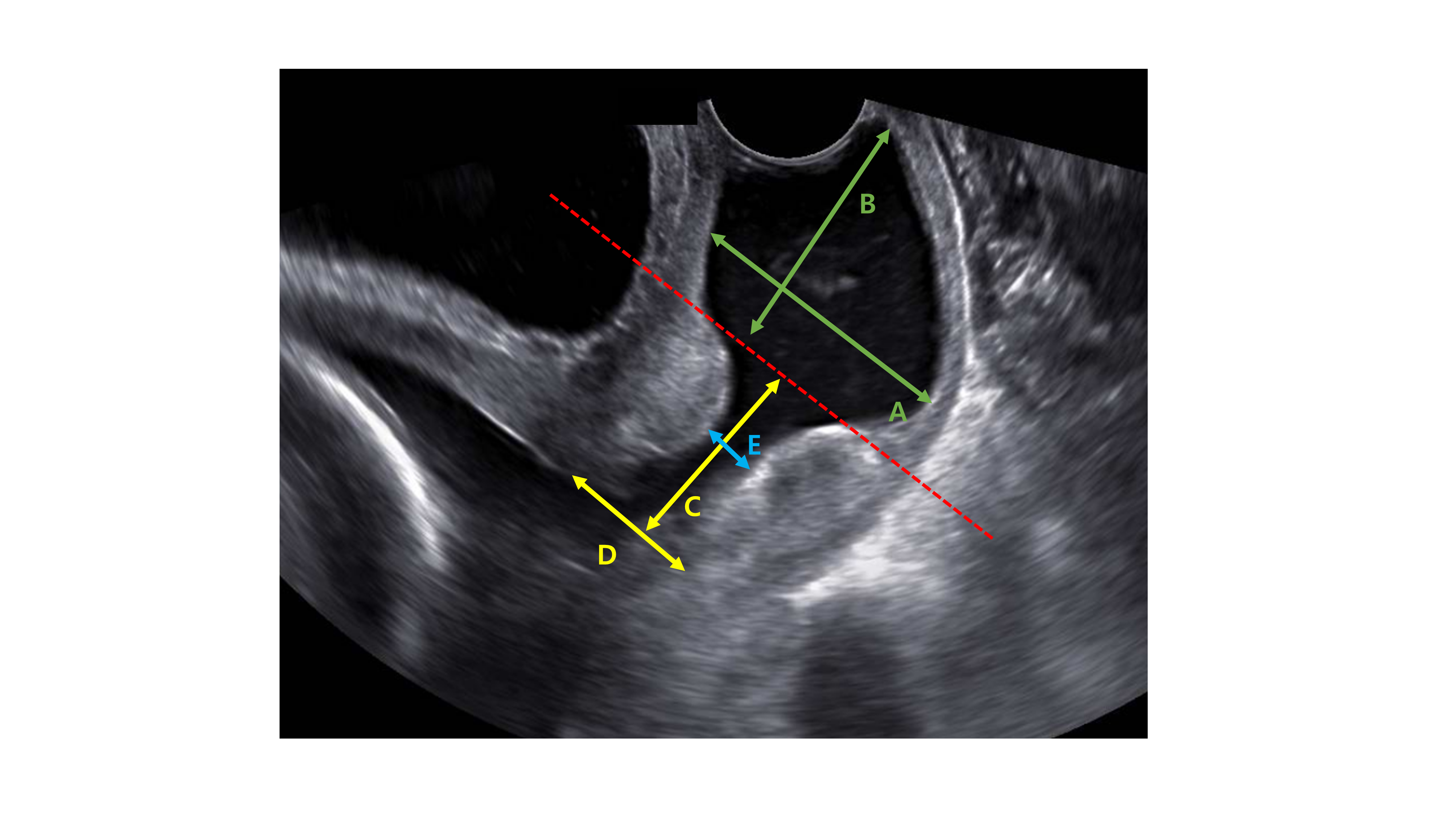

Supplement: Supplementary file 1 [file jcm-12-02480-s001.zip › jcm-2273923-supplementary.tif]
